# A Preoperative Diagnostic Nomogram to Predict Tumor Subclassifications of Intrahepatic Cholangiocarcinoma

**DOI:** 10.3390/cancers17101690

**Published:** 2025-05-17

**Authors:** Mizuki Yoshida, Masahiko Kinoshita, Yuta Nonomiya, Ryota Kawai, Ayumi Shintani, Yasunori Sato, Takahito Kawaguchi, Ryota Tanaka, Shigeaki Kurihara, Kohei Nishio, Hiroji Shinkawa, Kenjiro Kimura, Akira Yamamoto, Shoji Kubo, Takeaki Ishizawa

**Affiliations:** 1 Department of Hepato-Biliary-Pancreatic Surgery, Osaka Metropolitan University Graduate School of Medicine, 1-4-3 Asahimachi, Abeno-ku, Osaka 545-8585, Japan; miz828828@gmail.com (M.Y.); j22652b@omu.ac.jp (T.K.); taanaakaa3364@gmail.com (R.T.); s-kurihara0731@hotmail.com (S.K.); u21474n@omu.ac.jp (K.N.); d21129t@omu.ac.jp (H.S.); v21873r@omu.ac.jp (K.K.); kubosho65@yahoo.ne.jp (S.K.); take1438@gmail.com (T.I.); 2 Department of Medical Statistics, Osaka Metropolitan University Graduate School of Medicine, 1-4-3 Asahimachi, Abeno-ku, Osaka 545-8585, Japan; f21811t@omu.ac.jp (Y.N.); ryota.kawai@omu.ac.jp (R.K.); ayumi.shintani@gmail.com (A.S.); 3 Smart Data & Knowledge Services, Deutsches Forschungszentrum für Künstliche Intelligenz (DFKI), Trippstadter Straße 122, 67663 Kaiserslautern, Germany; 4 Department of Human Pathology, Kanazawa University Graduate School of Medical Sciences, 13-1 Takaramachi, Kanazawa, Ishikawa 920-8640, Japan; sato-ya@med.kanazawa-u.ac.jp; 5 Department of Radiology, Osaka Metropolitan University Graduate School of Medicine, 1-4-3 Asahimachi, Abeno-ku, Osaka 545-8585, Japan; akira@omu.ac.jp; 6 Health Education Course, Department of Education, Faculty of Education, Shitennoji University, 3-2-1 Gakuenmae, Habikino, Osaka 583-8501, Japan

**Keywords:** intrahepatic cholangiocarcinoma, subclassification, nomogram

## Abstract

Background/Objectives: Intrahepatic cholangiocarcinoma (ICC) is subclassified into small and large duct types. Although these subclassifications may help determine the appropriate treatment strategy, subclassification diagnosis currently depends on postoperative pathological examinations. This study aimed to establish a nomogram to predict ICC subclassifications. Methods: This study included 126 patients with ICC who underwent liver resection. The participants were divided into small and large duct-type ICC groups. A nomogram to predict large duct-type ICC was developed using four diagnostic imaging findings: rim-type enhancement in the early phase, an absence of tumor enhancement in the early phase, the presence of peripheral biliary dilatation due to tumor invasion, the presence of penetrating Glisson’s vessels in the tumor, and two laboratory test results: serum gamma-glutamyl transpeptidase and carbohydrate antigen 19-9 levels. Nomogram performance was also assessed. Moreover, the bootstrap method and calibration plots were used to assess nomogram validity. Results: Seventy and fifty-six patients were pathologically diagnosed with small and large duct-type ICCs, respectively. The area under the curve of the established nomogram was 0.93 and remained 0.91 after Harrell’s bias correction. The sensitivity and specificity of the nomogram developed using the Youden index were higher than those of any of the characteristic imaging findings. Calibration plots demonstrated a strong association between the nomogram and the actual data. Conclusions: We developed a novel preoperative nomogram to predict large duct-type ICC. This nomogram can be clinically useful for predicting the subclassifications of ICCs and may contribute to the establishment of a more appropriate treatment strategy for ICC.

## Simple Summary

Intrahepatic cholangiocarcinoma is subclassified into small and large duct types. The appropriate treatment strategy may differ between the small and large duct types because of clinicopathological differences. However, the subclassification diagnosis currently depends on postoperative pathological examinations. Therefore, we developed a nomogram to predict the subclassification of intrahepatic cholangiocarcinoma preoperatively using characteristic imaging findings and laboratory test results. The nomogram exhibited a high predictive performance; therefore, it can be clinically useful for predicting tumor subclassifications and establishing a more appropriate treatment strategy for intrahepatic cholangiocarcinoma.

## 1. Introduction

Intrahepatic cholangiocarcinoma (ICC) is the second most common liver malignancy after hepatocellular carcinoma. ICC is subclassified into small and large duct types according to the World Health Organization (WHO) Classification of Tumors, 5th edition [1]. Differences in the clinicopathological characteristics between large and small duct-type ICCs have been demonstrated. Large duct-type ICC is usually located in the perihilar region of the liver. It comprises mucin-producing columnar tumor cells with a large duct or papillary architecture and exhibits periductal infiltration (PI) or PI + mass forming (MF) [2,3]. In large duct-type ICC, the following risk factors have been identified: primary sclerosing cholangitis, hepatolithiasis, liver flukes, and exposure to chlorinated organic solvents (e.g., 1,2-dichloropropane) [1,4,5]. In addition, large duct-type ICC displays a prediction for lymph node metastasis (LNM) and is associated with a poor prognosis [3,6,7]. By contrast, small duct-type ICC is mainly located in the peripheral region of the liver, comprises mucin-poor cuboidal cells, forms small ductular or tubular structures, and exhibits MF [2,3]. The risk factors for small duct-type ICC include chronic liver disease, such as viral hepatitis [4], and small duct-type ICC has a relatively lower risk of LNM than large duct-type ICC [3,6,7].

Based on the clinicopathological differences between subclassifications, our recent study has suggested that the appropriate treatment strategy, especially the indications for lymph node dissection (LND), may differ between subclassifications [7]. In other words, the subclassification of ICC is a significant factor that contributes to determining the appropriate treatment strategy for ICC and ultimately improving treatment outcomes. However, although recent studies have reported the characteristic imaging findings of small and large duct-type ICCs [6,7,8], subclassification diagnosis is mainly based on postoperative pathological examination. Therefore, ICC subclassification is not commonly used to determine treatment strategies for ICCs. This is a significant clinical limitation. Subclassification has the potential to establish an appropriate treatment strategy; thus, it may have a significant clinical impact on the development of preoperative diagnostic tools for ICC subclassification.

This study aimed to establish a nomogram to predict the subclassification of ICCs preoperatively using several preoperative factors, including characteristic imaging findings.

## 2. Materials and Methods

### 2.1. Study Cohort and Ethical Statement

This retrospective study included 131 patients with ICC who underwent liver resection in our hospital between January 1998 and December 2022. Patients with unavailable data, including those who did not undergo preoperative dynamic computed tomography (CT), were excluded. Ultimately, 126 patients were included in this study.

This study was approved by the Ethics Committee of Osaka Metropolitan University (no. 2022-116) and conducted in accordance with the Declaration of Helsinki. Written informed consent was obtained from all patients.

### 2.2. Factors Included in the Nomogram

Our recent study, which investigated the differences in clinicopathological characteristics between small and large duct-type ICCs, revealed that serum gamma-glutamyl transpeptidase (γGTP) and carbohydrate antigen 19-9 (CA19-9) levels were significantly higher in patients with large duct-type ICC than in patients with small duct-type ICC [7]. On dynamic CT imaging, patients with small duct-type ICC were more likely to exhibit rim-type enhancement in the early phase, whereas those with large duct-type ICC demonstrated higher frequencies of absence of tumor enhancement in the early phase, the presence of peripheral biliary dilatation due to tumor invasion, and the presence of penetrating Glisson’s vessels in the tumor [6,7,8]. Based on these results, we developed a preoperative diagnostic nomogram to predict ICC subclassification including these six factors.

### 2.3. Pathological Examination

ICC subclassifications were pathologically diagnosed according to the WHO Classification of Tumors, 5th edition [1]. Subclassification was determined based on formalin-fixed paraffin-embedded tissue sections stained with hematoxylin and eosin (H&E). Mucin-producing columnar cells were identified as histopathological features of large duct-type ICC. In contrast, mucin-poor cuboidal cells represented the histopathological features of small duct-type ICC. In 96 of the 126 cases, the H&E staining results combined with the primary location and gross features of the ICC enabled the subclassification diagnosis. In cases where subclassification could not be determined based on the H&E staining results (n = 30), immunohistochemical analysis was performed. The primary antibodies against S100P (1:100 dilution, ab133554; Abcam, Cambridge, UK) and SPP1 (1:200 dilution, ab214050; Abcam), which have been identified as discriminatory markers for large and small duct-type ICCs, respectively, were used to determine the subclassification [9,10].

### 2.4. Diagnostic Imaging

**Figure 1:**
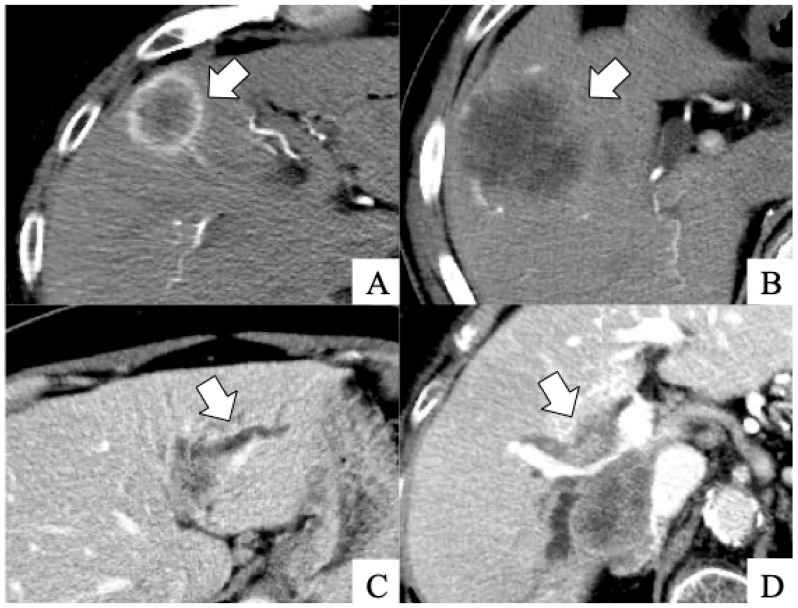
Dynamic computed tomography scan findings. (**A**) Rim-type enhancement in the early phase (arrow). (**B**) Absence of tumor enhancement in the early phase (arrow). (**C**) Peripheral biliary dilatation due to tumor invasion (arrow). (**D**) Penetrating Glisson’s vessel in the tumor (arrow).

Four clinical physicians (MY, MK, SK, and radiologist AY) retrospectively evaluated all the dynamic CT imaging results of the 126 patients. The following imaging results were investigated: rim-type enhancement in the early phase, the absence of tumor enhancement in the early phase, the presence of peripheral biliary dilatation due to tumor invasion, and the presence of penetrating Glisson’s vessels in the tumor. Figure 1 shows typical imaging results.

### 2.5. Statistical Analyses and Model Development

The Mann–Whitney U test was used to compare continuous variables between groups. Categorical variables were compared between the groups using the chi-squared or Fisher’s exact test. A logistic regression analysis was performed to determine the coefficient for each factor. The logistic regression model included the six preoperative factors: γGTP level, CA19-9 level, rim-type enhancement in the early phase, the absence of tumor enhancement in the early phase, the presence of peripheral biliary dilatation due to tumor invasion, and the presence of penetrating Glisson’s vessel in the tumor. Although CA19-9 often had extreme values, values > 10,000 were considered clinically acceptable, even if considered as 10,000, and were therefore set at 10,000. Furthermore, γGTP and CA19-9 values had a wide right-skewed distribution. To reduce skewness, the values were log-transformed before being inputted into the prediction model.

A receiver operating characteristic (ROC) curve was used to determine the cutoff value, sensitivity, and specificity of the prediction model based on the Youden index. To evaluate its predictive ability, a comparison of sensitivity and specificity between the model and each imaging finding was performed using log-binomial regression with a generalized estimating equation considering the exchangeable correlation structure. The discriminative ability of the prediction model was assessed using the area under the ROC curve (AUC). In addition, two regression models were developed. One included the four imaging findings (image model), and the other included log-transformed γGTP and CA19-9 values (laboratory model). The 95% confidence interval (CI) of the difference between the prediction and image models was estimated using 1000 bootstrap resampling. For the internal validation of the prediction model, the 95% CI of the AUC was estimated using the bootstrap method with 1000 iterations. The bias-corrected AUC of the prediction model was calculated using Harrell’s bias correction. Calibration curves were constructed to assess the predictive performance of the regression model. A nomogram was developed to represent the logistic regression model graphically. Statistical analyses were performed using the R Statistical software (version 4.3.2; R Core Team 2023). A two-sided *p*-value < 0.05 was used to indicate a statistically significant difference.

## 3. Results

### 3.1. Differences in Characteristics Between Small and Large Duct-Type ICCs

In this cohort, 70 patients were pathologically diagnosed with small duct-type ICC, and 56 patients with large duct-type ICC. Table 1 shows the differences in the characteristics of small and large duct-type ICCs.

No significant differences in the median age of the patients or the chronic liver disease rates, including that of viral hepatitis, were observed between the small and large duct-type ICC groups. The two laboratory test result comparisons revealed significantly higher serum CA19-9 levels in the large than in the small duct-type ICC group. The median value of serum γGTP levels was higher in the large than in the small duct-type ICC group, without any significant difference. On dynamic CT imaging, no significant differences in the tumor size between the two groups were observed between the two groups. Rim-type enhancement in the early phase was significantly more common in the small duct-type ICC group. In contrast, an absence of tumor enhancement in the early phase, the presence of peripheral biliary dilatation due to tumor invasion, and the presence of penetrating Glisson’s vessels in the tumor were significantly more common in the large duct-type ICC group.

### 3.2. Preoperative Diagnostic Nomogram to Predict the Subclassifications of ICCs

**Figure 2:**
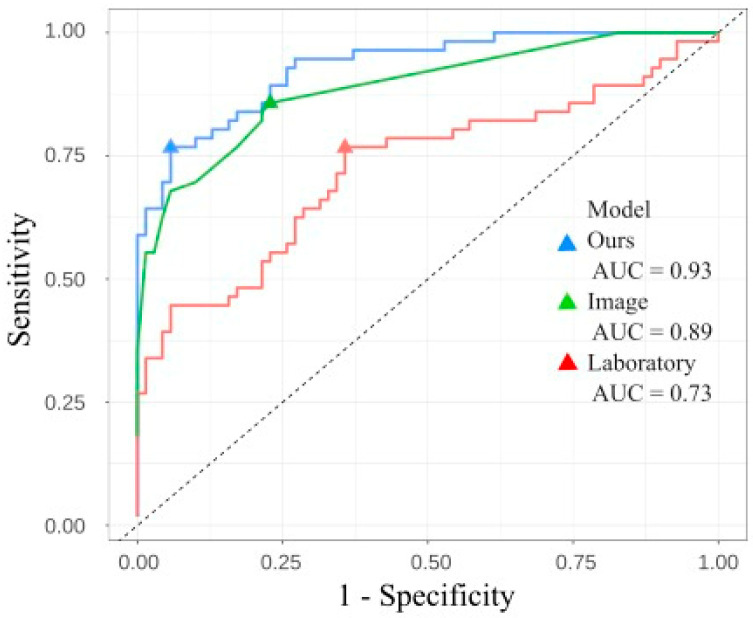
Comparison of receiver operating characteristic ROC curve between our prediction, image, and laboratory models. AUC, area under the ROC curve.

A logistic regression model to preoperatively predict the large duct-type ICC was developed, including the six factors: rim-type enhancement in the early phase, the absence of tumor enhancement in the early phase, the presence of peripheral biliary dilatation due to tumor invasion, the presence of penetrating Glisson’s vessel in the tumor, and the log-transformed values of γGTP and CA19-9 with reference to our recent study [7]. The ROC curve of the model including these six factors showed an AUC of 0.93 (95% CI, 0.89–0.97) (Figure 2).

**Figure 3:**
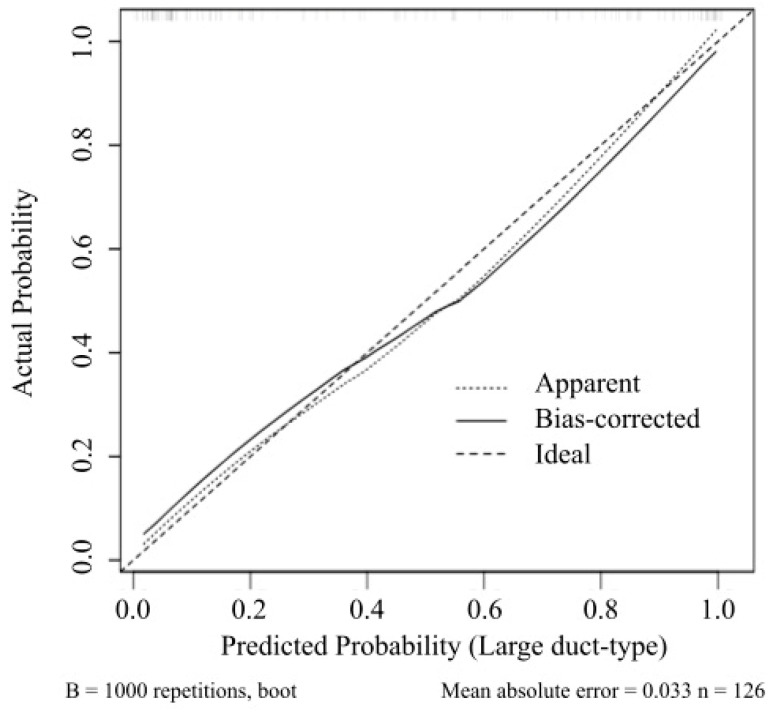
The calibration curve for assessing the accuracy of the nomogram. Model accuracy is visualized by comparing predicted versus actual probabilities of large duct-type intrahepatic cholangiocarcinoma, showing the apparent predictive ability and bias correction for overfitting. The relative prevalence of probability levels is indicated by the vertical lines at the top of the plot.

We also developed two other regression models: the image model, which included the four imaging findings, and the laboratory model, which included the log-transformed values of γGTP and CA19-9. Figure 2 depicts the ROC curve and AUC of the regression model (0.93; 95% CI, 0.89–0.97), the image model (0.89; 95% CI, 0.83–0.94), and the laboratory model (0.73; 95% CI, 0.63–0.82). The results revealed that the regression model exhibited superior performance compared with the other models. The 95% CI of AUC by bootstrapping was 0.89–0.97. Similarly, the difference between the prediction and image models estimated using the bootstrap method was 0.041 (95% CI, 0.017–0.092). The bias-corrected AUC was 0.91. The calibration line is shown in Figure 3, where the line from the prediction model closely resembles the ideal line, indicating favorable predictive accuracy between the actual and predicted probabilities.

**Figure 4:**
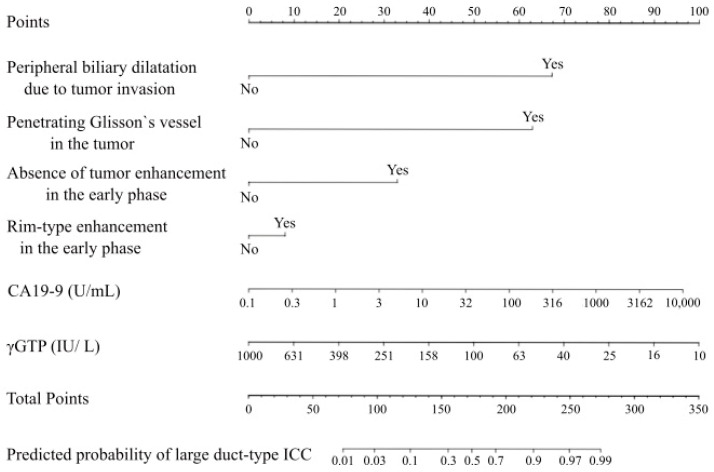
Nomogram to predict large duct-type ICC. γGTP, gamma-glutamyl transpeptidase; CA19-9, carbohydrate antigen 19-9.

Based on the six preoperative factors, a nomogram was developed to predict large duct-type ICC (Figure 4).

### 3.3. Sensitivity and Specificity Using the Developed Nomogram

The optimal cutoff value of the model was 0.64, which corresponded to the Youden index, with a sensitivity of 77% and a specificity of 94%. The sensitivity and specificity of the model were compared for the single imaging findings (Table 2).

The model demonstrated significantly higher sensitivity and specificity than no rim-type enhancement in the early phase (77 vs. 59%, *p* = 0.049; 94 vs. 73%, *p* = 0.003). Moreover, the sensitivity of the model was significantly higher than in the absence of tumor enhancement in the early phase (77 vs. 32%, *p* < 0.001). The sensitivity and specificity of both peripheral biliary dilatation due to tumor invasion and penetrating Glisson’s vessels in the tumor were lower than those of the developed model, without significant differences (77 vs. 70%, *p* = 0.40; 94 vs. 89%, *p* = 0.24).

## 4. Discussion

In the present study, a preoperative diagnostic nomogram was developed to predict large duct-type ICC using four diagnostic imaging findings and two laboratory test results. The nomogram demonstrated high predictive ability, with an AUC of 0.93, which remained at 0.91 after Harrell’s bias correction. The nomogram was further validated internally using the bootstrap method, and the calibration plot showed good predictive accuracy. The subclassification of ICCs, which is currently mainly diagnosed postoperatively, can be accurately predicted by a diagnostic tool using several preoperative clinical factors.

Several studies have attempted to identify the differences between subclassifications using preoperative imaging findings [6,8]. Although the size of the tumor does not differ between small and large duct-type ICCs, enhancement patterns and biliary abnormalities are useful for predicting tumor subclassifications [6,7,8]. In large duct-type ICC, the diffuse arrangement of fibroblasts and collagenous stroma associated with Glisson fibrous capsules leads to an absence of tumor enhancement in the early phase [6,11,12]. In addition, because cholangiocarcinoma develops in the large bile ducts and progresses into the liver parenchyma, peripheral biliary dilatation and penetrating Glisson’s vessels can be detected in large duct-type ICC [6,7,8]. In small duct-type ICC, the fibrous stroma is distributed at the tumor center and is surrounded by peripheral tumor cells, leading to rim-type enhancement in the early phase [11,12]. However, the sensitivity and specificity of each of these characteristic imaging findings are insufficient for a highly accurate preoperative subclassification. The nomogram developed in the present study demonstrated significantly higher sensitivity and specificity than the characteristic imaging findings, which are the primary factors in the preoperative subclassification diagnosis.

In this cohort, although the univariate logistic regression analysis showed that the small duct-type ICC group was more likely to exhibit rim-type enhancement in the early phase, as in previous reports [6,7,8], the multivariate analysis to develop the nomogram showed the opposite result. The reasons for this result, in addition to the small number of cases in the cohort, include the relatively low sensitivity and specificity of rim-type enhancement in the early phase in our study cohort, which is similar to the findings of a previous report [6]. Moreover, although the diagnostic imaging findings were evaluated by four experts in the present study, it is sometimes difficult to detect rim-type enhancement in the early phase, especially in small tumors. This might have influenced our present results, and further investigation with a larger number of patients is required to reveal the association between rim-type enhancement in the early phase and ICC subclassifications. However, it is difficult to identify the subclassification of ICC using single imaging findings. Therefore, an analysis using a combination of several clinical findings is essential for an accurate preoperative subclassification based on our present results.

Clinicopathological differences in ICC subclassifications have been demonstrated in some reports [3,8,13,14]. In our recent study, we suggested that hepatectomy with LND and/or biliary reconstruction should be considered in patients with large duct-type ICC, whereas hepatectomy without these advanced procedures, including laparoscopic surgery, should be considered in patients with small duct-type ICC, based on clinicopathological differences between subclassifications [7]. At present, the indications for and clinical significance of LND for ICC remain controversial [7,15,16,17,18]. One reason for this is that existing studies contain mainly mixed analyses of small duct-type ICC, which has a low frequency of LNM, and large duct-type ICC, which has a high frequency of LNM. The incidence of viral hepatitis, a known risk factor for small duct-type ICC [7,8,13], differs between Western and Asian countries [19,20]. The proportion of each subclassification in the ICC population may differ by country or region, possibly contributing to inconsistent study results. Although the effect of LND in each subclassification needs to be verified, the subclassification may play an important role in setting appropriate indication criteria for LND because of the differences in LMN frequency. In this context, preoperative diagnosis using the nomogram proposed in the present study may significantly contribute to the establishment of preoperative criteria for LND.

Previous reports have indicated that chronic hepatitis, including viral hepatitis, is a risk factor for small duct-type ICC [7,8,13]. However, no significant difference was observed in the proportion of patients with chronic viral hepatitis between the small and large duct-type ICC groups in the present study. This is partly because this study was retrospective and conducted at a single institution. Additionally, the sample size was relatively small and may have been influenced by the high incidence of hepatitis viruses in the area surrounding the hospital. Based on these cohort biases, the present study did not include background liver disease to develop a nomogram. However, the developed nomogram achieved high predictive performance without a history of background liver diseases, including viral hepatitis. Moreover, the diagnostic accuracy of the subclassification may be further improved by considering background liver diseases, including viral hepatitis, in addition to our highly accurate nomogram.

This study has some limitations. First, this was a single-center retrospective study with a small cohort. Therefore, the nomogram was not validated in this validation cohort. In addition, patient prognosis and the incidence of LNM have not been evaluated because of this background. However, unexpectedly, a combination of imaging findings and laboratory test results was used to develop a highly accurate preoperative diagnostic nomogram despite the small cohort of patients. Therefore, this nomogram should avoid using a similar method through larger multicenter prospective studies to enable a more accurate preoperative diagnosis, and it has the potential to predict the prognosis and LNM of ICCs in combination with other prognostic factors. Second, the nomogram did not include magnetic resonance imaging (MRI) findings because the participants in this cohort did not always undergo MRI. Recent studies have demonstrated the value of MRI in predicting the subclassification of ICC [8,12,21], and there is the potential to improve the predictive accuracy of the model using MRI findings. An external validation study should be conducted soon to confirm the usefulness of the developed nomogram.

## 5. Conclusions

We developed a novel preoperative nomogram to predict large duct-type ICC. This nomogram may be clinically useful for predicting the subclassification of ICC and may contribute to the establishment of a more appropriate treatment strategy for ICC.

## Figures and Tables

**Table 1:** Characteristics of small and large duct-type ICC.

Variables	Patients with Small Duct-Type ICC (n = 70)	Patients with Large Duct-Type ICC (n = 56)	*p*-Value
Age, years	68 (32–89)	70 (42–82)	0.79
Sex, male: female, n	52:18	32:24	0.066
Chronic liver disease, n	39	25	0.29
Viral hepatitis (HCV: HBV), n	18 (14:4)	13 (11:2)	0.91
Alcoholism, n	11	3	0.088
MASH, n	11	11	0.73
Hepatolithiasis, n	0	3	0.085
Laboratory test results, median (IQR)			
Total bilirubin level, mg/dL	0.6 (0.3–22.7)	0.6 (0.2–1.6)	0.72
Albumin level, g/dL	4.2 (3.2–4.8)	4.1 (3.2–4.9)	0.66
AST level, U/L	30 (11–164)	26 (16–160)	0.28
ALT level, U/L	26 (7–208)	21 (11–148)	0.64
γGTP level, IU/L	63 (13–951)	104 (15–620)	0.11
Log(γGTP), log(IU/L) *	4.1 (2.6–6.9)	4.6 (2.7–6.4)	0.11
Prothrombin activity, %	99 (59–150)	102 (68–142)	0.17
Platelet count, ×10^4^/μL	18.7 (5.4–32.8)	20.1 (5.9–51.1)	0.57
CRP level, mg/dL	0.10 (0.01–7.12)	0.22 (0.01– 8.5)	0.088
CEA level, ng/mL	3.4 (0.7–56.9)	4.1 (0.6–2108.5)	0.070
CA19-9 level, U/mL	21 (2.0 –1554)	146.5 (0.1–10,000)	<0.001
Log(CA19-9), log(U/mL) *	3.0 (0.7–7.4)	5.0 (0.7–9.2)	<0.001
Dynamic CT scan findings			
Rim-type enhancement in the early phase, n	51	23	<0.001
Absence of tumor enhancement in the early phase, n	4	18	<0.001
Peripheral biliary dilatation due to tumor invasion, n	8	39	<0.001
Penetrating Glisson’s vessel in the tumor, n	8	39	<0.001
Tumor size, cm	3.9 (0.4–12.5)	3.7 (0.8–13.0)	0.81
Median (interquartile range)			

ICC, intrahepatic cholangiocarcinoma; HCV, hepatitis C virus; HBV, hepatitis B virus; MASH, metabolic dysfunction-associated steatohepatitis; AST, aspartate aminotransferase; ALT, alanine aminotransferase; γGTP, gamma-glutamyl transpeptidase; CRP, C-reactive protein; CEA, carcinoembryonic antigen; CA19-9, carbohydrate antigen; and CT, computed tomography. * The values are displayed on a natural logarithmic scale.

**Table 2:** Diagnostic performances of single imaging findings and the nomogram for large duct-type ICC.

Imaging Finding	Sensitivity	*p*-Value ^a^	Specificity	*p*-Value ^b^
Developed nomogram, %	77%		94%	
No rim-type enhancement in the early phase, %	59%	0.049	73%	0.003
Absence of tumor enhancement in the early phase, %	32%	<0.001	94%	1.00
Peripheral biliary dilatation due to tumor invasion, %	70%	0.40	89%	0.24
penetrating Glisson’s vessel in the tumor, %	70%	0.40	89%	0.24

The sensitivity and specificity of the developed nomogram were calculated using the Youden index. Diagnostic performances of the imaging findings and the nomogram for large duct-type ICC were described. ^a^ *p* values were obtained by comparing sensitivity between the developed nomogram and the single imaging finding. ^b^ *p* values were obtained by comparing specificity between the developed nomogram and the single imaging finding.

## Data Availability

The original contributions presented in this study are included in the article. Further inquiries can be directed to the corresponding author.

## Abbreviations

The following abbreviations are used in this manuscript:

γGTP	Gamma-glutamyl transpeptidase
AUC	Area under the ROC curve
CA19-9	Carbohydrate antigen 19-9
CI	Confidence interval
CT	Computed tomography
H&E	Hematoxylin and eosin
ICC	Intrahepatic cholangiocarcinoma
LND	Lymph node dissection
LNM	Lymph node metastasis
MF	Mass forming
PI	Periductal infiltration
ROC	Receiver operating characteristic
WHO	World Health Organization
